# Repeatable Territorial Aggression in a Neotropical Poison Frog

**DOI:** 10.3389/fevo.2022.881387

**Published:** 2022-04-27

**Authors:** Sarah Chaloupka, Mélissa Peignier, Susanne Stückler, Yimen Araya-Ajoy, Patrick Walsh, Max Ringler, Eva Ringler

**Affiliations:** 1 Department of Behavioral and Cognitive Biology, University of Vienna, Vienna, Austria; 2 Messerli Research Institute, University of Veterinary Medicine Vienna, Vienna, Austria; 3 Division of Behavioral Ecology, Institute of Ecology and Evolution, University of Bern, Bern, Switzerland; 4 Centre for Biodiversity Dynamics, Department of Biology, Norwegian University of Science and Technology, Trondheim, Norway; 5 School of Biological Sciences, Institute of Evolutionary Biology, University of Edinburgh, Edinburgh, United Kingdom; 6 Department of Evolutionary Biology, University of Vienna, Vienna, Austria; 7 Institute of Electronic Music and Acoustics, University of Music and Performing Arts Graz, Graz, Austria

**Keywords:** territoriality, aggression, animal personality, poison frogs, *Allobates femoralis*

## Abstract

Intra-specific aggressive interactions play a prominent role in the life of many animals. While studies have found evidence for repeatability in boldness, activity, and exploration in amphibians, we know relatively little about consistent among-individual variation in aggressiveness, despite its importance for male-male competition and territoriality. Amphibians, and Neotropical poison frogs (Dendrobatidae) in particular, are highly suitable for investigating among-individual variation in aggressiveness, as most species exhibit strong territoriality in at least one of the sexes. In the present study, we aimed to fill this gap in knowledge, by investigating within- and between-individual variation in territorial aggression in a semi-natural population of the Neotropical poison frog *Allobates femoralis* (Dendrobatidae) in French Guiana. We conducted repeated, standardized behavioral tests to assess if the level of territorial aggression is consistent within and different between individuals. Further, we tested a possible link between body size and level of territorial aggression. We found moderate repeatability in territorial aggressiveness, but no link to age and/or body size. In conclusion, our study represents the first documentation of repeatable aggressive behavior in a territorial context in amphibians.

## Introduction

An increasing number of studies have investigated within-individual consistency and between-individual variation of behavior over time and across contexts, termed animal personality (Réale et al., 2007; Dingemanse et al., 2010; Zidar et al., 2017; Goursot et al., 2019). Animal personality is typically characterized along five main axes, including activity, aggressiveness, boldness/shyness, exploration/avoidance, and sociability (Réale et al., 2007). These axes constitute behavioral traits that affect multiple behaviors of an organism in specific contexts. Theoretical models and empirical research have shown that behavioral differences, along any axis, can affect individual fitness through their effects on survival and mating success (Sih et al., 2004a,b; Smith and Blumstein, 2008; Amy et al., 2010; Réale and Dingemanse, 2010; Sih et al., 2012; Wolf and Weissing, 2012).

Several studies recently highlighted that frogs, toads, newts, and salamanders display repeatable behaviors along at least three personality axes: boldness, activity, and exploration (reviewed in Kelleher et al., 2018). Interestingly, no study to date has focused on the aggressiveness axis in amphibians, despite its importance for male-male competition and territoriality. This might be due to the focus so far being on aquatic species and/or species from temperate regions that commonly do not establish territories. However, aggressiveness is common in many amphibians where males defend and fight over resources or territories (Mathis et al., 1995; Wells, 2007; Dyson et al., 2013). This behavior is particularly well-known in Neotropical poison frogs (Dendrobatidae, *sensu* AmphibiaWeb, 2021) who offer ideal prerequisites to study within- and between-individual variation in aggressive behavior (e.g., Duellman, 1966; Summers and Amos, 1997; Summers, 2000; Gardner and Graves, 2005; Poelman and Dicke, 2008; Galeano and Harms, 2016; Gonzáles-Santoro et al., 2021; for a review see Pröhl, 2005).

Here, we use the Brilliant-Thighed Poison Frog *Allobates femoralis* (Aromobatinae) to investigate within- and between-individual variation in male aggressiveness in the context of territorial defense. This species occurs across the Amazon Basin and Guiana Shield in local disjunct populations. Males are highly territorial during the reproductive season and announce their territory to male competitors and female mating partners *via* a prominent advertisement call broadcasted from elevated structures like fallen branches or logs (Hödl et al., 2004). Females are not territorial but display site fidelity to perches from where they travel to male territories for courtship and mating (Ringler et al., 2012; Fischer et al., 2020). Territorial males approach and aggressively repel calling conspecifics that intrude into their territory, as territory possession is a prerequisite for male mating success in *A. femoralis* (Narins et al., 2003; Montanarin et al., 2011; Ringler et al., 2011; Ursprung et al., 2011a; Stückler et al., 2019).

Although featuring individually distinct calls (Gasser et al., 2009), territorial *A. femoralis* have been shown to react aggressively to familiar neighbors and strangers in playback experiments, probably because during the reproductive season territory intrusions by either neighbors or strangers are equally threatening to the territory holder (Tumulty et al., 2018). A recent study further found that younger individuals were more likely to attack a non-threatening model (i.e., mimicking a female or a non-calling male) during acoustic playback than older, more experienced frogs, indicating the importance of experience and learning for identifying and distinguishing potential mating partners from rivaling individuals (Sonnleitner et al., 2020). Given that territorial advertisement and defense are costly in terms of energy expenditure and risks of predator exposure (Ryan et al., 1983; Taigen and Wells, 1985; Pough and Taigen, 1990; Wells, 2007; Dyson et al., 2013), we expect that the corresponding costs-benefits trade-off should lead to the emergence of the personality trait “aggressiveness” in males. Males featuring high levels of aggressiveness might be better at defending their territory, but might suffer from increased energy expenditure, risk of injury or predation. Males that show lower levels of aggression might in turn benefit from higher survival chances until the next season. Accordingly, we would expect to observe within-individual consistency and between-individual differences in aggressive responses to acoustic playbacks mimicking a territory intruder.

## Materials and Methods

### Ethics

This study was approved by the scientific committee of the “Nouragues Ecological Research Station” and the ethics board of the University of Veterinary Medicine Vienna. Behavioral experiments were conducted in strict accordance with current French and EU law, following the Study of Animal Behaviour (ASAB) guidelines ASAB (2020). Permissions for working and sampling were provided by the CNRS Guyane (“Centre National de la Recherche Scientifique Guyane”), and by the “Ministère de la Transition Écologique et Solidaire” (permit number: TREL2002508S/303).

### Study Population and Area

The study was conducted in a free living population of *A. femoralis*, located on a ~5 ha river island in the vicinity of the field camp “Saut Pararé” of the CNRS Nouragues Ecological Research Station (4°02′ N, 52°41′W; WGS84), within the nature reserve “Les Nouragues” in French Guiana (Bongers et al., 2001; Ringler et al., 2016). The population was established in 2012 by introducing 1,800 tadpoles to the island, where *A. femoralis* had not occurred previously (Ringler et al., 2014). Since then, a stable adult population of about 150 individuals has established, and detailed information about genetic relatedness, body size and age of all individuals is available from a long-term monitoring of the island population.

We conduced daily surveys of individuals in the study population to assess and monitor their distribution and movements. Every frog was photographed on scale paper from its dorsal- and ventral-side for later individual identification and assessment of body size. We recorded the exact location, sex, picture numbers, and current activity of each frog on a high-resolution background map of the island (Ringler et al., 2016), using tablet PCs (WinTab 9, Odys, Willich, Germany) and the mobile GIS software ArcPad 10.2 (ESRI, Redlands, CA, United States). Individuals were sexed based on the presence (males) or absence (females) of vocal sacs. For individual identification, we compared the pictures of the individually unique ventral patterns using the pattern matching software Wild-ID (Bolger et al., 2012). To assess individual body sizes, we measured snout-urostyle length with the aid of a scale paper using the software ImageJ (Rasband, 1997-2021).

### Quantification of Aggressive Behavior

**Figure 1:**
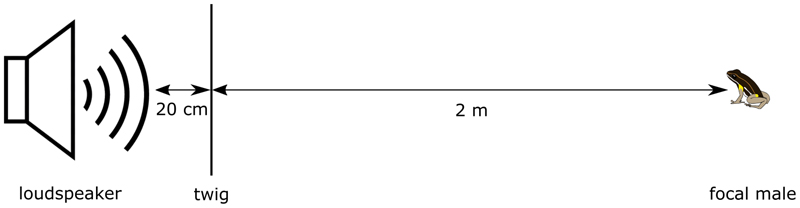
Experimental setup to assess individual aggressiveness of territorial *A. femoralis* males.

For the present study, we measured the level of aggressiveness as the agonistic response of an individual toward a simulated conspecific male entering its territory (Réale et al., 2007). During agonistic encounters, male *A. femoralis* typically orientate their head/body, jump toward the intruder and wrestle (Hödl, 1987; Narins et al., 2003). We simulated a territory intruder by broadcasting a standardized synthetic advertisement call from a loudspeaker with an integrated music player (MUVO 2c, Creative, Singapore) positioned 2 m from and facing the focal male (Figure 1). A twig from the forest floor was positioned 20 cm in front of the speaker as a perimeter marker for a successful approach to the speaker. After placing the speaker, we waited 2 min before starting the playback to take a suitable position to conduct the trial. The synthetic call featured the average spectral and temporal parameters of another free-ranging population of *A. femoralis* in French Guiana based on recordings by Gasser et al. (2009); for a detailed description see Ursprung et al. (2009) and Ringler et al. (2017). The playback contained 25 bouts of 10 calls each, with equally long interbout-intervals, totaling a duration of 6 min 42 s, and was presented from WAV-files (16-bit, 44.1 kHz). We calibrated the speaker once per week with a sound pressure meter (SL-100, Voltcraft, Hirschau, Germany) to produce the playback signal at a sound-pressure level (SPL) of at least 69 dB (re 20 μPa; A, fast) at a 2 m distance, which lies within the range of natural variation in this species and considered to be the minimum threshold for eliciting a positive phonotactic response in *A. femoralis* (Hödl, 1987).

During the playback, the experimenter stayed approximately 2 m behind the speaker and documented the movements of the focal male using a voice recorder (ICD-PX240, Sony, Tokyo). We recorded the following behaviors during the trial: first head-body orientation (“head”), first jump toward the speaker (“jump”), and when the frog crossed within 20 cm of the speaker (“finish”). Trials were scored as successful when either the frog came within 20 cm of the speaker or when the playback signal ended. We scored trials as unsuccessful and excluded from the analyses when the focal male began calling during the playback, as this can be interpreted as the intruder/speaker being outside of the defended area of the male’s territory (Ringler et al., 2011). Both behaviors, antiphonal calling as well as phonotactic approach, can be interpreted as “aggressive” territorial behaviors, but in our experiment we only focused on the phonotactic response as a measure of territorial aggression.

After the trials we captured the focal frog and took ventral and dorsal images for identification. To account for local variation in sound transmission, we then measured the SPL of the playback stimulus at the initial location of the focal frog, with the speaker in the same location as for the trial. We successfully tested 32 individual males, and replicated tests four times, with a minimum of 7 days between two consecutive trials to minimize habituation effects to the experimental setup.

From the audio recordings we then extracted the latencies from the start of the playback until the first head-body orientation, until the first jump, and until the arrival within 20 cm of the speaker using the software Audacity 2.2.1 (Audacity Team, 1999-2017). We further determined the approach speed over 1.8 m from the time between the first jump and the arrival within 20 cm of the speaker (cf. Figure 1). Individuals who did not react to the speaker were not given a threshold value to avoid right or left censoring.

### Statistical Analysis

All statistical analyses were conducted R v3.6.0 (R Core Team, 2020) using the integrated development environment RStudio v1.3.1093 (R Studio Team, 2019). The data generated and the code used for analyses are available at the Open Science Framework (link for review purposes: https://osf.io/fdxqm/?view_only=9427c034f20f466f99d30fdd1397752e). To investigate the prevalence of an aggressiveness personality trait in *A. femoralis*, we calculated the adjusted repeatability of the measured behaviors, as the proportion of phenotypic variation that can be attributed to between-subject variation (Nakagawa and Schielzeth, 2010).

First, we transformed the data that deviated from normality. We used the function “transformTukey” to apply a constant transformation on the latency until the first head-body orientation, until the first jump, until arrival to the speaker and on the speed to reach the speaker. We calculated repeatability from four linear mixed effect models with the function *lmer* in the lme4 package (Bates et al., 2016), each with one of the transformed behaviors as the response variable. A behavior was considered as repeatable when the 95% confidence intervals did not overlap zero. For each model, we included the SPL, but also the trial number, and the individual size as fixed effects, to account for habituation and effect of body size on aggressive responses. Previous studies have shown that age influences the accuracy of aggressive responses in *A. femoralis*, and that body size is positively correlated to age (Ursprung et al., 2011b; Sonnleitner et al., 2020). In all models ID was included as random effect to account for repeated measurements. We inspected model residuals for normal distribution using diagnostic qq-plots. Finally, we calculated the confidence interval using the function “confint.” The model featuring the latency until the first jump failed to converge with all possible optimizers, and therefore is excluded from our analysis.

## Results

Males took on average 27.2 s (±70.6 SD) to orientate their body toward the speaker and 43.8 s (±85.8 SD) to first jump toward the speaker. They took 85.5 s (±47.3 SD) on average to reach the speaker, with a mean speed of 3.8 cm s^−1^ (±2.4 SD).

**Figure 2:**
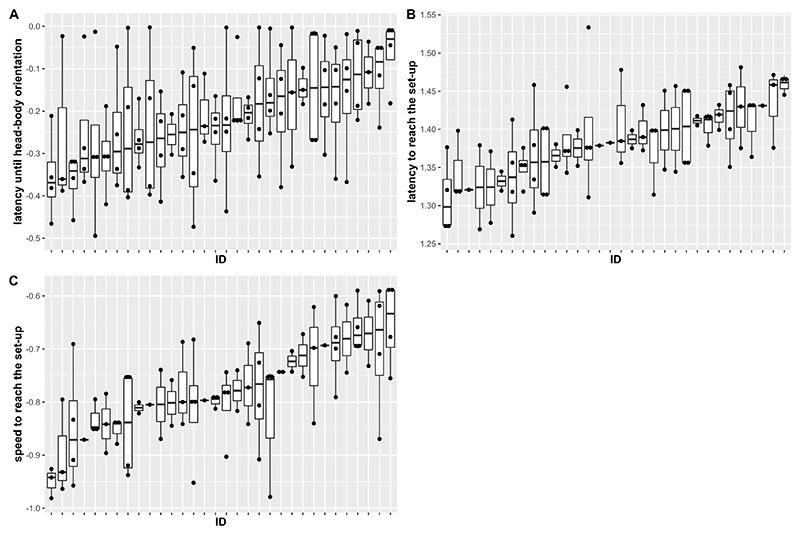
Latency to first head-body orientation **(A)**, latency to arrive at the speaker **(B)**, and speed to arrive at the speaker **(C)** for each of the 32 individual males. All variables have been transformed using a constant transformation. Males are ordered by amount of individual variation. For each individual, the dots represent the results of each of the trials in which the individual reacted to the speaker, while the horizontal bold line represents the median across these four trials. The upper and lower horizontal lines delimiting the boxes represent the first and third quartiles.

We found that phonotactic approach speed of *A. femoralis* males was repeatable (*R* = 0.37, 95% CI = 0.22–0.45). When looking at the measurements, a highly repeatable behavior would show low within-individual variability but high between-individual differences. In our case, individuals were indeed rather consistent in their speed, while between-individual variation was large (Figure 2). However, we did not find evidence for repeatability in the latency to perform a head-body orientation or the latency to arrive at the speaker (respectively, *R* = 0.07, 95% CI = 0–0.18 and *R* = 0.23, 95% CI = 0–0.33). Individuals were highly variable in the time they took to perform a head-body orientation, resulting in low among-individual variation and low within-individual consistency and therefore low repeatability (Figure 2). Conversely, individuals were more consistent in the time they took to reach the speaker, but between-individual variation was still low, resulting in a low repeatability (Figure 2). Finally, we observed that individuals reached the speaker quicker in the last compared to the first trial (Table 1, *p* = 0.028 and *p* = 0.016). Body size and SPL were not related to any behavioral measurement (Table 1).

## Discussion

We designed a standardized *in situ* experiment to collect data on the agonistic response of an individual toward a simulated conspecific intruding its territory, to evaluate its applicability for population-wide studies on animal personality in terrestrial anurans. Despite the small sample size in this study, we found repeatable differences among individuals in the speed to reach the speaker, suggesting the existence of personality along the aggressiveness axis in *A. femoralis*. These values lie in the range of average repeatability scores previously reported in behavioral studies in amphibians (*R* = 0.24–0.39; Brodin et al., 2013; Carlson and Langkilde, 2013; Urszán et al., 2015; Kelleher et al., 2017), and our choice of not censoring the data might have resulted in rather conservative estimates of repeatability.

In *A. femoralis*, high levels of aggression could entice an individual to react fiercer toward a conspecific intruding its territory or help a male to take over another territory. This is particularly relevant because the possession of a territory by a male is a prerequisite for reproductive success (Ursprung et al., 2011a). Aggressive individuals will, however, probably be more likely to engage in energetically costly and potentially physically harmful fights. Future studies should investigate if levels of aggressiveness are ultimately linked to the chance of winning or losing a territorial conflict, and also how this is related to individual fitness.

We did not find that the initial latency to respond to a conspecific intruder was repeatable. We see two possible explanations for this result: First, the lack of repeatability could be due to local variation in habitat complexity (i.e., vegetation density, leaf litter, and perch height) at the specific location where each respective trial was performed. Indeed, spectral degradation and reverberation have been found as important cues for acoustic distance assessment in *A. femoralis* (Ringler et al., 2017). Secondly, the absence of repeatability for the latency to respond to a conspecific intruder could be the result of a cost-benefit trade-off of aggressive responses to intruders. The latency to respond to an intruder might contain two discrete behaviors: an evaluation of the circumstances (i.e., own breeding status, known neighbors, etc.) and the decision, based on evaluated risks and benefits, of whether to make an aggressive approach. Once the decision to approach is taken, the individual level of aggressiveness takes over and drives the speed to approach the conspecific intruder, therefore leading to repeatable results throughout trials. Future studies should investigate how the different behaviors emitted in a given context are structured into one or several functional units (i.e., personality traits) using structural equation modeling (Araya-Ajoy and Dingemanse, 2014).

Simulating a territory intruder by broadcasting a standardized call proved to be a powerful tool to repeatedly measure aggressive response of male *A. femoralis*. Phonotactic experiments with simulated advertisement calls of conspecifics are a very common method to study anuran behavior, for example to measure territory borders of territorial amphibians (Narins et al., 2003; Rojas et al., 2006; Ringler et al., 2011), female preferences for male call traits (Tárano and Herrera, 2003; Akre and Ryan, 2010), or acoustic properties for species discrimination (Schwartz, 1987; Bee et al., 2001). However, such playback experiments had never been applied to assess repeatability of aggressive responses in individual territory holders. In the present study we used the same playback for all trials to assess individual consistency of territorial aggression in the same context. Future studies should use a random set of advertisement calls featuring a range of different spectral and temporal acoustic parameters to better assess aggressive responses across contexts.

While animal personality has been broadly documented in mammals, birds, fish and even invertebrates (e.g., Bell, 2005; Dochtermann and Jenkins, 2007; Tremmel and Müller, 2013; Zidar et al., 2017; Goursot et al., 2019), unfortunately we only have limited knowledge about within and between-individual behavioral variation in amphibians (but see Kelleher et al., 2018). This is surprising, as amphibians might provide key insights on the evolution of animal personality and its link to physiology, morphology and ecology, as they face extreme shifts in their ecological niche when they undergo metamorphosis (Wilson and Krause, 2012). Also many amphibians are territorial and vigorously defend their territories against conspecific intruders (Wells, 2007), offering ample opportunities to investigate the link between male-male competition and individual fitness. The present study is the first to investigate the prevalence of repeatable among-individual differences in territorial aggressiveness in free-ranging terrestrial neotropical anurans.

## Figures and Tables

**Table 1:** Mixed effect model results.

	Latency until head-body orientation		Latency to reach the speaker		Speed to reach the speaker	
**Fixed effects (estimate ± SE |*p*-value)**						
(Intercept)	−0.39 ± 0.45	0.383	1.46 ± 0.23	**<0.01**	−.02 ± 0.42	**0.015**
Trial	0.01 ± 0.01	0.403	−0.01 ± 0.01	**0.028**	0.02 ± 0.01	**0.016**
size	0.13 ± 0.13	0.313	0.03 ± 0.07	0.667	−0.03 ± 0.13	0.809
dB	−0.00 ± 0.00	0.459	−0.00 ± 0.00	0.304	0.00 ± 0.00	0.186
**Random effects (estimate ± SD)**						
ID	0.00 ± 0.03		0.00 ± 0.03		0.00 ± 0.06	
Residual	0.02 ± 0.13		0.00 ± 0.05		0.01 ± 0.08	

The estimates, standard-error and p-values are presented. Significant results (p-value < 0.05) are written in bold.

## Data Availability

The datasets presented in this study can be found in online repositories. The names of the repository/repositories and accession number(s) can be found below: https://osf.io/fdxqm/?view_only=9427c034f20f466f99d30fdd1397752e.
